# 9th German Conference on Chemoinformatics

**DOI:** 10.1186/1758-2946-6-S1-I1

**Published:** 2014-03-11

**Authors:** Uli Fechner

**Affiliations:** 1 GDCh-CIC Division Associated Board Member, Beilstein-Institut zur Förderung der Chemischen Wissenschaften, Trakehner Str. 7–9, 60487 Frankfurt, Germany

## 

The Chemistry-Information-Computer (CIC) division of the German Chemical Society (GDCh) [1] invited the chemoinformatics and molecular modelling community to the 9^th^ German Conference on Chemoinformatics (GCC2013) to discuss recent developments in the field of Computational Chemistry. The conference was held from the 10^th^ to the 12^th^ November 2013 in Fulda, Germany.

It was the first time for the current CIC board, who took office in the beginning of 2013, to organize the German Conference on Chemoinformatics. The board decided to adapt the conference to the recent developments in Computational Chemistry and the novel challenges the field is faced with. The session *Chemical Information*, *Patents and Databases* made room for the session *Hot Topics and Developments*, the scientific focus of other sessions was shifted, and a novel session, *Research Telegrams*, provided PhD Students an opportunity to present the current status of their research in oral presentations of 15 minutes each. Furthermore, new members were welcomed to the Scientific Advisory Board and the conference venue was moved from Goslar, Germany, where the conference has been held for the last eight years, to Fulda, Germany.

The 26 oral presentations covered Molecular Modelling and Drug Design, Chemoinformatics, and Materials Science. 62 posters were presented in two poster sessions. The more than 140 attendees from 20 nations demonstrated that the German Conference on Chemoinformatics is an internationally well-established event in the global Chemoinformatics and Modelling community.

**Figure 1:**
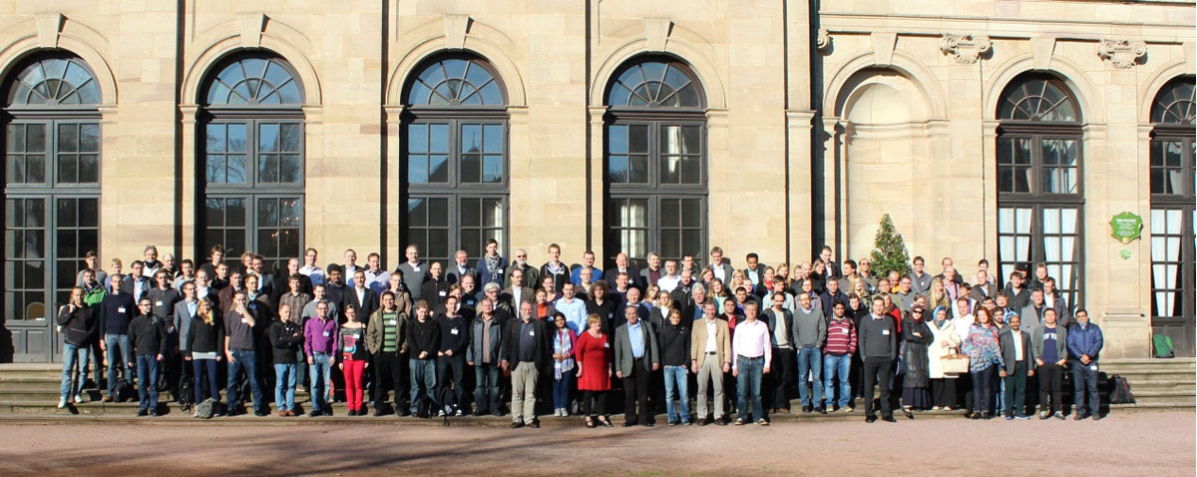
Participants of the 9th German Conference on Chemoinformatics (GCC2013), November 10–12, 2013 in Fulda, Germany.

The CIC-Award for Computational Chemistry 2013 for the best PhD thesis in the realm of Computational Chemistry was granted to Dr. Anne Mai Wassermann for her excellent PhD thesis "Computational Analysis of Structure-Activity Relationships – From Prediction to Visualization Methods", which she worked on under the supervision of Prof. Dr. Jürgen Bajorath at the Rheinische Friedrich-Wilhelms-Universität Bonn, Germany [2].

**Figure 2:**
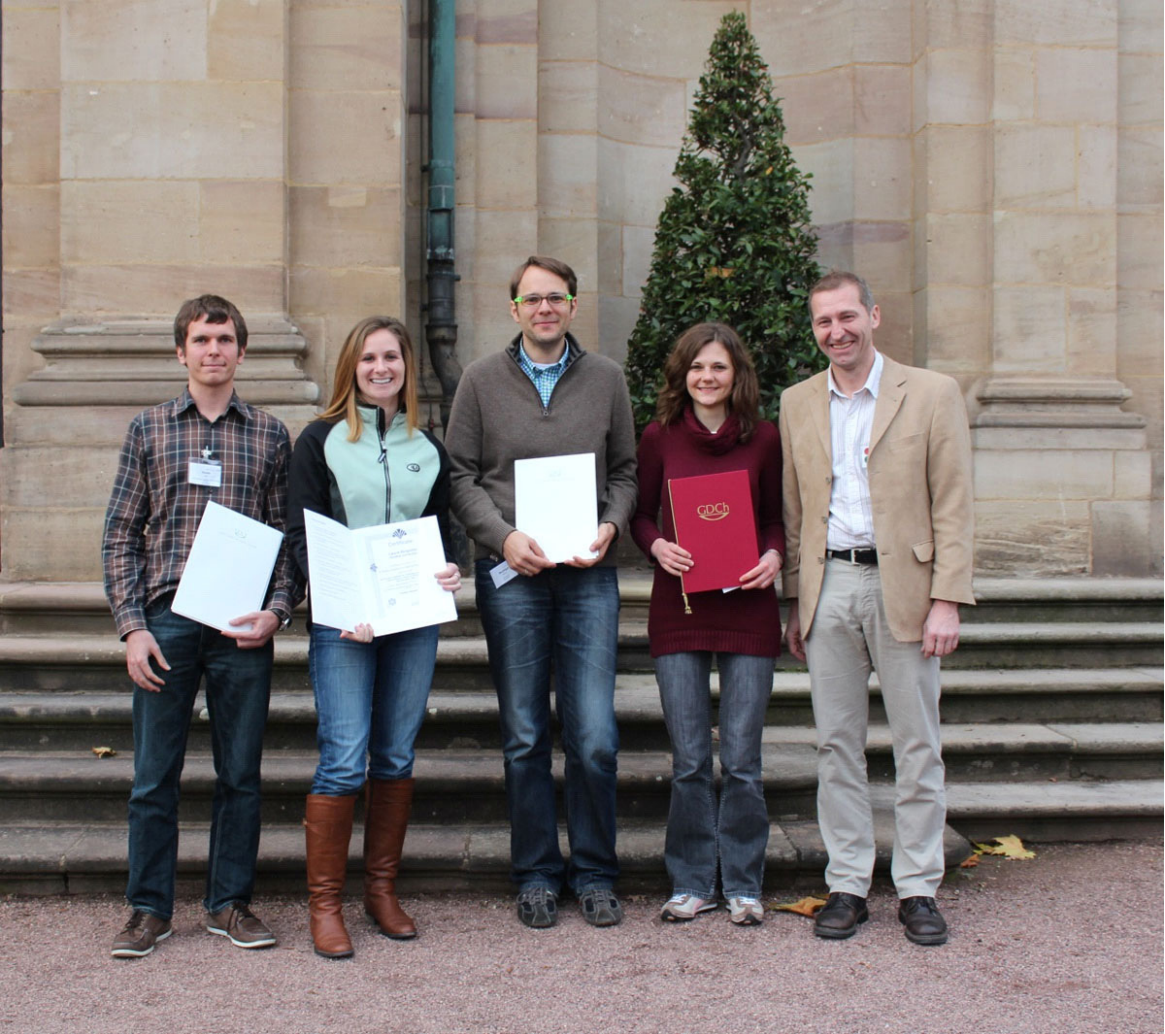
The winners of the poster award and the CIC-Award for Computational Chemistry 2013: from left to right, Jens Kunze (poster award, Swiss Federal Institute of Technology, Zurich, Switzerland), Laura J. Kingsley (poster award, Purdue University, West Lafayette/IN, USA), Michael Reutlinger (poster award, Swiss Federal Institute of Technology, Zurich, Switzerland), Anne Mai Wassermann (CIC-Award for Computational Chemistry, dissertation prize; Novartis Institutes for Biomedical Research, Cambridge/MA, USA) and Thomas Engel (Chair of the GDCh CIC division).
